# Guidelines for support to mothers of sexually abused children in North-West province

**DOI:** 10.4102/curationis.v40i1.1689

**Published:** 2017-07-25

**Authors:** Gaboipolelwe M. Masilo, Mashudu Davhana-Maselesele

**Affiliations:** 1 Department of Nursing Science, North-West University, South Africa

## Abstract

**Background:**

South Africa is reported to have the highest rate of sexual assault in the world with over 40% of cases occurring among children. Children who are sexually abused have support programmes and policies to assist them in coping, but there are no support programmes for mothers or caretakers. Caretakers need support for themselves and assisting them will incrementally benefit children under their care. Often mothers of these children experience shock, anger, disbelief and suffer secondary trauma such as depression and post-traumatic stress disorder (PTSD) following their children’s sexual abuse disclosure and yet there are no guidelines for support to these mothers within North-West province (NWP)

**Objectives:**

The study seeks to develop guidelines for support to mothers of sexually abused children in NWP.

**Methods:**

Concurrent convergence triangulation mixed method design was employed in this study. The population consisted of mothers of sexually abused children (SAC) (*n* = 17 participants for the qualitative component and *n* = 180 participants for the quantitative component). A sample of mothers of SAC was purposely selected.

**Results:**

The participants indicated significant levels of depression because of lack of support by stakeholders. Guidelines for support to assist mothers cope with their secondary trauma were developed based on the literature review, study findings as well as an ecological model of the impact of sexual assault on women’s mental health. The results also showed extreme PTSD (47.8%), little support (38.8%), not coping (76.1%) and depression (36.1%).

**Conclusion:**

The stakeholders should consider a positive approach to support mothers whose children are sexually abused.

## Introduction

Child sexual abuse (CSA) is a crime committed on children across the globe, which makes mothers of sexually abused children (SAC) undergo psychological changes post-disclosure (Zimba 2013:1). Despite the fact that these mothers undergo psychological changes post-disclosure, it seems that there are no visible guidelines in place on how to support these mothers. The focus of CSA is only placed on children.

Jones (2011:8) concurs with Zimba (2013:1) that CSA disclosure affects mothers negatively, and they experience psychological and emotional trauma and as such they need support. Jones (2011:8) and Domian et al. (2010:403) mention that supporting mothers post-CSA disclosure is vital because they deal with the loss of their children’s virginity, family relationships and they also experience emotional as well as financial hardships, especially if they are not employed.

This is supported by Jackson (2008:12), who conducted a study in the United States titled ‘Forgotten Victims: Mothers of Sexually Abused Girls’, which revealed that mothers of SAC girls are traumatised because of CSA disclosure of their daughters and that the effects of CSA affect these mothers’ psychological well-being; therefore, they need both social and financial support to overcome the secondary trauma and pain caused by the disclosure of CSA. Jackson (2008:1) and Domian et al. (2010) report that mothers of SAC acknowledge that research on CSA focuses on the victim but not the treatment and support they require post-disclosure. Mothers’ lack of support post-disclosure makes them unable to support their children effectively and this delays the healing process.

The researcher conducted a comprehensive literature review on existing formal and informal support systems and programmes available for the mothers of SAC. The literature search provided the researcher with a comprehensive idea of research conducted to date regarding support programmes available to mothers of SAC. The articles were to describe a programme for support to mothers of SAC post-disclosure and acknowledge a need for the support system of a mother, experiences of the mother regarding the child’s sexual trauma, mothers’ levels of coping, depression, post-traumatic stress disorder (PTSD) post-disclosure and existing health care policies focusing on support for mothers of SAC.

The literature indicated that South Africa has the highest rates of sexual assault in the world and 20 000 cases of sexual assault and attempted rape are reported yearly involving children younger than 18 years and yet their mothers are not supported. The results indicate how serious the problem of CSA is throughout South Africa, especially in North-West province (NWP).

The results from the reviewed articles indicated that internationally only a few countries have developed programmes to support these mothers (Masilo 2011:14), but in African countries, there is a dearth of literature regarding support guidelines or programmes post-disclosure.

Based on the literature review, the researcher conducted a mixed methods design to explore and describe the experiences of mothers of SAC post-disclosure as well as determine the correlations between demographic data, coping strategies, levels of PTSD, depression and social support of these mothers. The data revealed that 43% of mothers of SAC suffered depression after CSA disclosure while 57% of them were not depressed post-disclosure. In addition the results showed that 87.8% had high levels of PTSD and 12.2% had low levels of PTSD after disclosure of CSA. These quantitative results concur with the qualitative results, where mothers experienced a range of emotions such as self-blame, suicidal ideation and psychological pain after finding out that their children were sexually abused (Willingham 2007:17). Mothers experienced PTSD after their children disclosed CSA; they blamed themselves for not noticing that sexual abuse was going on or that they had failed to protect their children. They felt that they should have known that the perpetrator was dangerous (Myrick & Green 2013:194). Furthermore, Myrick and Green (2013) reported that mothers usually feel stuck with the sense of danger and get disconnected from reality, which results in difficulty in managing daily situations.

Other factors that affected the mothers of SAC were finances, employment and marital relationships. In addition, some mothers of SAC presented with avoidance coping characteristics such as remembering the CSA disclosure (Myrick & Green 2013:194). These aforementioned signs and symptoms revealed by Myrick and Green (2013) are supported by Tavkar and Hansen (2011:189), who reported that mothers of SAC expressed significant levels of distress following CSA disclosure. They experienced elevated levels of stress, but a variety of distress symptoms were seen as evidenced by the mothers being restless (McMillan 2013).

The findings from the literature and mixed methods were utilised to develop guidelines for support with the aim of assisting professionals to render better care to mothers of SAC in NWP. The researcher’s view was that mothers of SAC are unseen and voiceless people whose experiences regarding their CSA disclosure must be heard so that supportive guidelines could be developed to assist policymakers and professionals to sufficiently address their problems, especially in NWP.

## Problem statement

SAC have policies to assist them in coping with the sexual abuse, but their parents who support them tend not to have any such support. Research conducted by Tavkar and Hunsen (2013:6) acknowledges that existing literature focuses only on assessing whether mothers support their abused children after disclosure and yet little is known about their challenges and the type of support they receive post-CSA disclosure.

The current study investigates the effects of CSA disclosure, specifically the ecology of the mothers psychologically, socially and spiritually because they do not seem have any guidelines that support them in their traumatic experiences. It was therefore necessary to develop guidelines that would assist those in close contact with mothers who have SAC in NWP.

## Purpose

The purpose of this study was to develop guidelines that would assist stakeholders to support and empower mothers of SAC in NWP to cope with secondary trauma experienced following CSA disclosure.

## Objectives of the study

The specific objective of the study was to describe the guideline that would be used to support mothers of SAC in NWP following disclosure.

### Significance

The research findings of this study would assist policy developers to ensure implementation of guidelines for mothers of SAC following CSA disclosure. The research findings for this study will also assist healthcare providers to inform curriculum developers on providing training for professionals. How significant is this to the mothers?

### Definition of concepts

#### Support

Support is the act of sustaining life by food or providing a means of subsistence or activity of contributing to the fulfilment of need or furtherance of an effort or purpose (Simpson & Speake 1998:1440). In this study, it means giving emotional, psychological, spiritual, financial and physical care to mothers of SAC following sexual abuse disclosure by family members, clinicians and the legal system.

#### Experience

Experience is a practical contact with and observation of facts or events (Simpson & Speake 1998:501). In this study, experience refers to what is happening in the lives of non-offending mothers of SAC, after the disclosure of sexual abuse of the child, that is, happening in her, and the changes within her, that is, psychologically, physically and socially.

#### Mother of sexually abused children

A mother is a woman in relation to the child or children to whom she has given birth (Simpson & Speake 1998:928). It includes any non-offending woman, stepmother, caregiver, foster parent, adoptive parent, grandparent and biological mother who is taking care of the SAC aged 0–16 years.

#### Child

According to *Children’s Act No. 38 of 2005*, a child means any person under the age of 18 years. In this study, it is a person, either a male or female, of any race under the age of 16 years, who was sexually abused within 12 months prior to the commencement of data collection.

#### Child sexual abuse

According to the Republic of South Africa (RSA 2007:7), CSA is an unlawful and intentional act of actual penetration without consent of the complainant. In this study, it is forced sexual intercourse with a child who is 16 years old and below without the consent of one of the parties.

#### Sexual abuse disclosure

Disclosure is to make new information known (Simpson & Speake 1998:408). In this study, it denotes the period after CSA activity is revealed.

### Research strategy

The study was divided into three phases, with phase 1 consisting of two stages. Stage 1 was qualitative while stage 2 was quantitative. In stage 1, the experiences of mothers of SAC were explored and described while in stage 2, coping skills, social support, depression and PTSD of mothers of SAC were examined following disclosure of their children’s sexual abuse. Phase 2 offers a literature review and provides a conceptual framework while phase 3 focuses on guidelines development, conclusion, limitations and recommendations.

## Research context

### Research design

The concurrent triangulation mixed method was employed by using both qualitative and quantitative design within the same research process (Creswell 2009:67). There were two relatively independent stages, one qualitative and the other quantitative. The two data sets were merged for an overall interpretation so that a complete picture could be obtained from both data sets (Creswell & Clark, 2007).

The population comprised non-abusive mothers of SAC and participants aged 19–70 years whose children were aged 0–16 years. A purposive sampling technique was used to select mothers of SAC. In-depth unstructured interviews were conducted with 17 participants until data saturation was reached. In stage 1, Tesch’s method was used to analyse the data. The sample size for stage 1 was 17 participants, who were included with the 180 participants of stage 2 determined through the use of Raosoft sample size calculator. Four instruments, namely Brief Cope Inventory, Post-Traumatic Stress Disorder, Beck Depression Inventory and Social Support Grid, were used to collect data regarding coping, PTSD, depression level and social support level. Data were analysed using Statistical Package for Social Sciences (SPSS version 21). Chi-square and frequency distribution were employed to analyse the demographic characteristics and examine the association among depression, PTSD levels, coping strategies and social support received of participants.

### Ethical consideration

The researcher obtained ethical clearance from the North-West University Research Ethics Committee (NWU-00010-12-A9). Permission to conduct the study was obtained from the North-West provincial department and informed consent was obtained from the participants. The participants were informed that they were free to withdraw from participation any time (Polit & Beck 2010:127) and were made aware that their withdrawal from participation would not prevent them from receiving any service. The names of the participants were protected, and confidentiality and privacy were maintained.

## Results

The findings showed that mothers expressed various types of trauma. They also blamed themselves for not taking care of their children and demonstrated a wide range of psychological difficulties and lack of support from stakeholders. The results also showed extreme PTSD at 47.8%, little support calculated at 38.8%, not coping up to 76.1% and little depression pegged at 36.1%. There was no statistical significance among the variables because the *p*-values were more than the significance level of 0.05. For instance, PTSD and social support *p*-value was 0.363, depression and social support *p*-value was 0.540, coping skills and PTSD *p*-value was 0.576 and coping and depression *p*-value was 0.648.

Ecological model of the impact of sexual assault on women’s mental health was used as a guide to assess the risk factors that could impede or promote the recovery process of these mothers.

**FIGURE 1:**
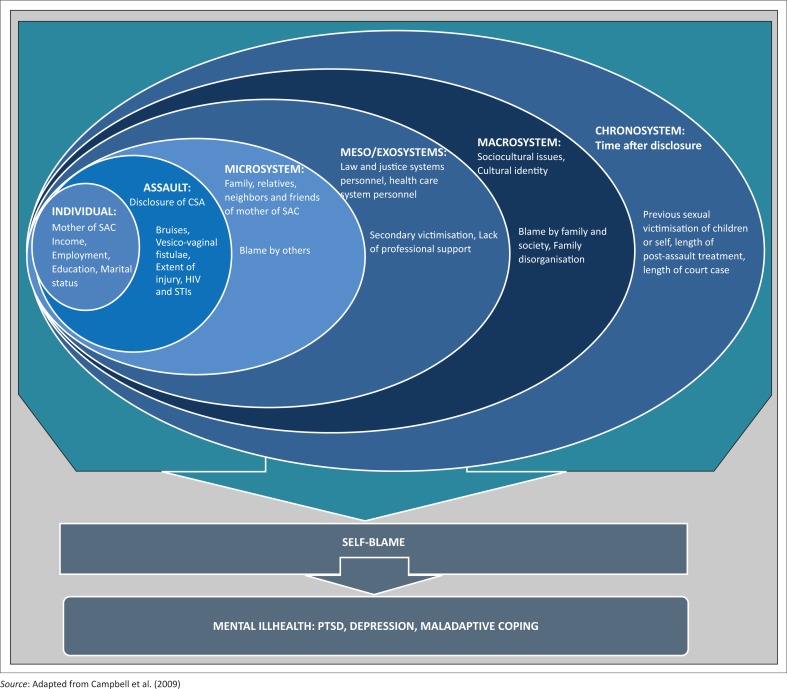
Ecological model of the impact of sexual assault on women’s mental health.

The literature revealed that these mothers experienced secondary trauma and consequently suffered from PTSD and depression (Willingham 2007:71). Therefore, their recovery process is a concern when diagnosed with PTSD and depression. Ecological model of the impact of sexual assault on women’s mental health developed by Campbell, Dworkin and Cabral (2009) identifies or lists the risk factors that could impede or promote the recovery process (Figure 1). The risk factors discussed are individual system, assault-related factors, microsystems, exosystem, mesosystem, macrosystem and chronosystem. This model addresses the changing ecological environment in which an individual resides and relationship at the larger social contexts that an individual finds herself in. The model also provides a supportive framework for understanding individuals exposed to psychological trauma. It is against this background that the researcher used this model to develop guidelines for support to mothers of SAC.

### Description of the process of guidelines formulation

The researcher integrated the qualitative findings with quantitative findings with the view to explain or interpret qualitative results with quantitative results, to discuss the integrated results with the literature as well as to develop a conceptual framework based on the results of the study. The qualitative data collection focused on the exploration and description of experiences of mothers of SAC while the quantitative data addressed examination of the correlation between depression, social support, coping skill and PTSD. It was apparent from the study that mothers of SAC lack social support from stakeholders and exhibited symptoms of depression, PTSD and maladaptive coping. The lack of support necessitated the researcher to develop the guidelines based on the findings as well as the theoretical framework.

### Guidelines

The guidelines below were developed to enable the stakeholders to plan and create an ecological environment in clinical and societal settings and coordinate a set of multispectral interventions to respond positively to CSA.

### Individual-level factors

In the guidelines submitted here, the individual factors of ecological model of the impact of sexual assault on women’s mental health are evident. The five sociodemographic variables that can influence recovery of the mother of SAC are evident at the first level, namely personal factors, pre-existing mental health conditions, genetic and biological factors and coping responses.

### Sociodemographic variable

- Refer mothers of SAC to a psychologist and social worker for counselling on emotional, psychological and social support. Social support post-CSA reduces suicidal ideation, PTSD, depression and maladaptive behaviour.

### Personality factors

In these guidelines, the personality factors level is evident.

- Provide mother with information to prevent maladaptive behaviours, which involve certain levels of avoidance such as substance abuse, social isolation and disengagement. The maladaptive behaviour is associated with longer recovery period, higher levels of depression and PTSD.
- Provide mothers of SAC with adaptive strategies that help them to express their feelings about social support they receive. These adaptive strategies reduce stress and are related to faster recovery, low level of PTSD and depression. The social support received predicts adjustment.
- Encourage mothers of SAC to appreciate unconditional support provided to reduce emotional and psychological trauma of self-blame as well as to improve coping skills. Self-blame at this level is associated with PTSD and depression; if the mother is supported it takes a shorter time to recover from the secondary trauma. A one-stop centre should be made effective and accessible to all mothers of victims of sexual violence by stakeholders to support them, reduce the stigma attached as well as to regain from the lowered self-esteem. This helps them open up and talk and not to feel isolated or betrayed to work through panic disorders.
- Refer the mothers of SAC to psychologists, social workers and psychiatrists to deal with personality disorders. These professionals are to be aware of the personal factors so that during history taking they refer to relevant multidisciplinary teams.
- Professionals are to make mothers of SAC engage in self-introspection because this decreases levels of depression and anxiety. Individuals adjust easily if they are aware of their strengths and weaknesses.

### Pre-existing mental health conditions

- Refer mothers who had some depression prior to CSA disclosure or a suicidal history to psychiatric institutions. Pre-CSA disclosure depression may be associated with post-disclosure distress.
- Mothers who attempted suicide pre-assault experienced significantly more post-assault depression and anxiety such that they must be referred to relevant professionals for assistance more urgently than those who did not have pre-existing conditions.

### Genetic and biological factors

This sociodemographic variable was not applicable in these guidelines.

### Coping responses

- Provide mothers of SAC with emotional support and coping orientation over time to deal with stress. Coping orientation deals with stress, especially in a supportive environment such as social support from the family, friends, neighbours, colleagues and other stakeholders. The lack of social support is likely to make people use avoidance coping, thereby increasing the level of PTSD symptoms.

### Assault-related factors

The assault level is evident and this shows that the model that guides the study is appropriately adapted.

Assault involves the type of psychological trauma sustained by the mother post-disclosure. It is thus recommended that support for mothers should focus on:

- Engaging community-based groups or nongovernmental organisations (NGOs) in identifying resources for child care. This assists the mother to overcome the psychological and emotional pain and trauma caused by disclosure of CSA.
- Providing unconditional support in managing the injuries incurred as a result of CSA. Emotional trauma and pain resulting from CSA disclosure is related to post-assault distress, PTSD and maladaptive coping.
  ■ Post-CSA disclosure, mothers are to be provided with information on how to report the CSA, what is likely to happen during the process and how the injuries are to be treated. Lack of support and information could predispose a mother to present with depressive symptoms.
- The professionals must provide the mothers of SAC with support and counselling sessions post-disclosure of CSA. Counselling reduces maladaptive behaviour, depression, PTSD as well as making the environment responsive.

### Microsystem factors

Family, friends, peers and significant others are positive at this level of the model. Friends, family, peers and significant others must support mothers of SAC by encouraging them to talk about the trauma with those who they can trust because communication and interaction with friends facilitate emotional healing. Peer support moderates the relationship between traumatic stress and PTSD.

- Families and friends must provide intensive family support and parenting interventions to the mothers who need support. Family members should stop being judgmental and instead support mothers and their children.

### Mesosystem or exosystem factors

The professionals and community involve the following formal and informal systems: the legal system, the medical system, the mental health system and the advocacy community such as chaplains, community counsellors, community leaders, traditional healers, community developers, community organisations and NGOs. These professionals and communities are positioned at the third level of the ecological model of the impact of sexual assault on women’s mental health at the mesosystem or exosystem level. In order to render support to mothers of SAC:

- All professionals must help mothers to regain control in situations where they are helpless by giving them practical information on what to do in case their children are sexually abused such as not bathing the child and keeping the underwear unwashed to facilitate the assessment and forensic procedures that should be undertaken, controlling the turmoil and giving information that will assist the mother to regain self-concept, body image, self-identity and self-esteem.
- Professionals must formulate support group platforms where the mothers of SAC are enabled to vent emotions and talk about their fears so that they can develop coping skills. Venting prevents two types of self-blame: for the negative event as well as blaming one’s own actions.
- Forensic nurses must provide crisis intervention initiatives for mothers of SAC post-disclosure to prevent depression and maladaptive coping.
- Debriefing sessions must be employed by the resident psychologist in Thuthuzela Care Center to ensure that mothers whose children have sustained injuries during sexual abuse receive support to reduce the impact of trauma and flashbacks that are experienced by mothers of SAC. This psychological intervention reduces psychological pain, anxiety and suicidal ideation.
- Police officials must display positive attitudes and facilitate a caring environment when attending to mothers of SAC because negative attitudes hinder therapeutic effects expected for recovery from trauma, disbelief and shock.
- Police and judiciary must collaborate with sexual violence working groups to establish strategies that reduce obstacles in arresting and prosecuting perpetrators.
- Traditional leaders in the community programmes ought to teach about and analyse retrogressive traditional and cultural values.
- Communities ought to be aware that rape, sexual violence and any other form of gender-based violence are criminal acts.
- The chaplain must reinforce spiritual beliefs and prayer to the mothers because collaboration and self-directedness and being prayerful have been found to be an adaptive form of coping. Religion is an important aspect of life for the majority of people in South Africa and the chaplains must listen to mothers who mourn their losses and be patient with their emotional status. It is important to give spiritual support to a person who is emotionally hurt.
- Traditional leaders must be involved in community programmes in teaching mothers of SAC to analyse traditional and cultural values that often stigmatise mothers of SAC, such as seeking permission from other family members before laying charges against the perpetrator.
- Community counsellors and community health workers must be trained on how to render services to mothers of SAC: crises intervention services, short- and long-term rape counselling and social support.
- Community-based groups or NGOs must engage communities in identifying resources to support the mother in overcoming the psychological trauma caused by the child’s physical and emotional injuries.
- Community-based groups must identify resources that may contribute to providing protection and support to mothers of SAC.
- Advocacy campaigns by organisations and institutions should be implemented to share information with the entire society such as school teachers and the mother’s workplace.
- The government must provide enough resources that support and sustain mothers of SAC.
- The curriculum in educational institutions must include basic sensitisation, competency in identifying mothers of SAC who have lacked support post-disclosure and facilitate competency in medico-legal rape examination and victim support.
- Education institutions must include in all their curriculum programme modules about the support of mothers of SAC post-disclosure or gender violence issues.
- Universities and nursing colleges must offer forensic nursing courses in their programmes. Forensic nursing equips nursing students with counselling skills to identify a person who needs forensic nursing assistance.
- Colleges and universities should also offer short courses for other stakeholders: a community equipped with skills and knowledge related to forensic nursing and gender violence meets the needs of mothers of SAC.

### Macrosystem factors

This is the macrosystem level of the model that is represented by culture-specific beliefs, such as those that condone male violence against women. This makes the model that guides the study critically relevant in shaping the guidelines.

- The stakeholders must make the community aware that rape, forced prostitution, sexual violence and gender-based violence are criminal acts that must be reported to the police.
- The traditional leaders must be involved in the community programmes to teach them to analyse and choose the traditional and cultural values that often stigmatise the mother, such as asking permission from other people such as family members before laying charges against the perpetrator.
- Community culture-sensitive information should be disseminated to all service providers. This assists those who are providing culture-specific intervention programmes for mothers of SAC from different cultures.

### Chronosystem factors

In these guidelines, the chronosystem factors of the ecological model of the impact of sexual assault on women’s mental health are evident. Time is critical in this domain.

- Ensure legislation protecting CSA is not compounded by failure of criminal justice system taking too long to convict the perpetrator or court proceedings taking long thus leading to mother’s depression.
- Policymakers must adhere to guidelines that are nationally developed to support mothers of SAC and ensure timeous quality services in Thuthuzela Care Center.
- Policymakers must include management of mothers whose children have been sexually abused in the primary health care package. The following must be included in the package: identification and management of mothers of SAC, secondary victimisation at the clinical services, trauma because of lack of support and families that discourage the mother to report and lay charges against perpetrators of rape.

## Discussion

Age did not influence the level of depression, PTSD and anxiety, but these afflictions were found to be general in all age groups. Evidence shows that mothers of SAC suffered more depression when the child was under the age of 14 years.

The employment status or income level was associated with negative effects of CSA disclosure. Those who were economically challenged experienced increased stress post-CSA disclosure, so did mothers who were unemployed. The distress post-CSA increased the psychological sequelae of the mother, thus leading to self-blame, depression and PTSD. The level of education was neither seen to be influencing the reactions of mothers of SAC post-disclosure nor was it seen to be associated with post-CSA PTSD and depression.

Ethnicity, race and social class were not an influence on the recovery process of the mother of SAC post-disclosure. Mothers of SAC who were anxious developed more distress post-CSA disclosure.

Some mothers of SAC who had previous experience of sexual abuse were found to have a pre-existing mental health condition, which was attributed to the sexual assault of the child and prolonged justice system processes. These mothers suffered heightened depression, anxiety, self-blame and PTSD that were associated with CSA disclosure (Pretorius, Chauke & Morgan 2011).

No genetic or biological factors were identified as predictors of post-CSA disclosure, distress and maladaptive behaviour. This concurred with the findings of the Campbell et al. (2009) model.

Mothers who received negative social support from stakeholders post-CSA disclosure presented with increased distress and they used avoidance coping and ended up with increased PTSD and maladaptive conditions. Maladaptive coping was evident in mothers of SAC isolating themselves from the family, staying at home when they were supposed to bring their SAC for follow-up visits.

At the individual-level factors, all aspects of the model emerged because the mothers of SAC expressed self-blame, anxiety and they were observed to be exhibiting signs of depression, PTSD and maladaptive coping post-CSA.

The supportive relationship from friends and peers was explored and described and it emerged that peers and friends do not always support mothers of SAC. The mother is labelled by friends, family members as well as peers for allowing the child to be sexually abused. Mothers of SAC were stigmatised by friends as unproductive. The mother also experienced rejection from family members because she laid charges against the family member who perpetrated CSA without consulting the family first. The mother is also labelled for rejecting the family norms and beliefs of gender inequality and male dominance. In this study, mothers blamed themselves because they felt they should not have accommodated their family members who violated the child sexually and the sexual cultural orientation made them to accept the blame from others. The self-blame made them suffer depression, PTSD and maladaptive coping.

The mothers of SAC experienced negative attitudes from professionals who did not provide them with the procedures to follow when the child is sexually abused. They regarded police officers as ineffective and unreliable because they failed to arrest the perpetrators and the judiciary released the perpetrators without trial. Such systems can magnify the mother’s feeling of powerlessness, shame, and guilt and self-blame, which lead to depression, PTSD and maladaptive coping.

### Assault-related factors

Campbell et al. (2009:230) highlight that the recovery of the victim of sexual assault is dependent upon who the perpetrator is. Sexual assault by a partner has been found to be a predictor of PTSD, depression and self-blame (Campbell et al. 2009:232). In this study, the assault is the disclosure of the CSA to the mother. The factors that could improve post-sexual abuse disclosure assault on the mother of a SAC are physical injuries and the extent of those injuries on the child and the relationship of the child to the perpetrator. The mother’s recovery process might affect her negatively if the perpetrator is a family member and the extent of an injury – which both lead to expensive surgical procedures.

The assault-related factors of the model were observed in this study by mothers who reported that their children were abused sexually by brothers and cousins. The mothers developed depression and maladaptive coping. The findings confirm those of the adapted model where women suffer PTSD post-sexual assault. In this study, the results revealed that 87.8% of mothers of SAC had suffered high levels of PTSD. In the qualitative stage of the study, mothers expressed that they experienced emotional pain, suicidal ideation or extreme symptoms of PTSD post-CSA disclosure. All mothers reported the presence of depressive symptoms post-CSA disclosure. About 76.1% reported that they were not coping post-CSA while only 23.9% indicated that they coped. In the qualitative phase, the mothers indicated that they isolated themselves, reported suicidal ideation and were observed to be tearful.

The results revealed that there is no relationship between PTSD, depression, coping strategies and self-blame post-disclosure. The findings also indicate that maternal support and intervention are critical post-disclosure. The mothers blamed themselves for not protecting the child; they expressed that the type of injuries the children sustained increased their trauma, fearing that the child could contract HIV or AIDS. They experienced psychological pain, anxiety, maladaptive coping, symptoms of depression and PTSD. The researcher concluded that CSA must be immediately reported and treated.

At the relationship level, mothers indicated they had received various types of support from friends, peers and significant others; others were negative while a few had good interpersonal relationships. They also indicated having been blamed by husbands for not taking care of the children prior to CSA. Good interpersonal relationships improved the mother’s coping skills, decreased the level of depression and PTSD while negative interactions made her to be depressed, exhibit avoidance coping strategies as well as signs of PTSD.

In this study, the supportive relationship from friends and peers were explored and described and the model again reinforced the findings. The mother is labelled negatively by friends, family members as well as peers for allowing the child to be sexually abused. She is stigmatised by friends as a non-protective parent. The mothers also experienced rejection from family members because they laid charges against the family member who is a perpetrator without consulting the family first. In this study, mothers blamed themselves because they felt they should not have accommodated their family members who perpetrated crime. The self-blame made them to suffer depression, PTSD and maladaptive coping.

### Mesosystem or exosystem factors

According to Campbell et al. (2009:233), the recovery of the post-assault victims is negatively or positively affected by social support provided by social systems, such as police, medical care, mental health systems and community advocacy systems. Campbell et al. (2009:232) highlight that sexual assault is a predictor of PTSD and depression. In this study, the mothers of SAC experienced negative attitudes from professionals. They regarded police officers as unreliable because they failed to arrest the perpetrators and judiciary released the perpetrators without trial. If the mother does not receive these rehabilitative services, then such dereliction magnifies the mother’s feeling of powerlessness, shame and guilt and self-blame. Consequently, she could suffer depression, PTSD and maladaptive coping.

### Macrosystem factors

Campbell et al. (2009:233) report that research is very limited about macrosystem factors and that they could not find any difference in the post-rape symptoms. They did not find differences in their cultural attribution. The women attributed this phenomenon to cultural self-blame. The mother of SAC post-disclosure takes the blame for CSA on behalf of the perpetrator by blaming herself for not adhering to retrogressive cultural and traditional values and beliefs that instructed her not to lay charges against the perpetrator, especially if the perpetrator is a family member (Campbell et al. 2009:233). The recovery of the mother of SAC is thus negatively influenced by sociocultural normative practices.

Self-blame made the mother to suffer depression, PTSD, maladaptive coping as well as family disorganisation following CSA disclosure. The mothers stated that they had experienced lack of support through cultural beliefs that are imposed on them. These experiences made mothers angry and anxious. In this study, these factors indicated in the ecological model also emerged during data collection and analysis.

### Chronological factors

These include dimensions of time as they are related to the mothers’ environment. The elements in this system could be external, such as the time of disclosure and the history of previous assault (Campbell et al. 2009:233). The time of the woman’s previous sexual assault history could affect her mental well-being negatively. The woman may experience emotional pain or blame herself for being high when she was sexually assaulted or could be blamed by others. She could also present with anxiety, depression and PTSD symptoms as well as maladaptive coping. In this study, mothers of SAC whose other siblings were once abused sexually and whose court cases were not processed were more depressed than those whose court cases did not take long and whose children were abused without prior history of CSA.

## Conclusions

The guidelines for support to mothers of SAC were developed based on the literature reviewed and the ecological model of the impact of sexual assault on women’s mental health by Campbell et al. (2009). Guidelines were developed to enable stakeholders to plan and create an ecological environment in clinical and societal settings that facilitate a set of multi-sectorial interventions to respond to sexual abuse disclosure. These guidelines must be included in the in-service training programmes of both public and private hospitals, governmental and NGOs, community workers, lay counsellors and home-based caregivers so that they, in turn, can empower individuals and societies regarding support of mothers of SAC.
